# Development of a new screening method for faster kinship analyses in mass disasters: a proof of concept study

**DOI:** 10.1038/s41598-022-22805-w

**Published:** 2022-11-27

**Authors:** Sonia Kakkar, Phulen Sarma, Inusha Panigrahi, S. P. Mandal, Pankaj Shrivastava, R. K. Kumawat

**Affiliations:** 1 Department of Forensic Medicine, PGIMER, Chandigarh, 160012 India; 2 Department of Pharmacology, AIIMS, Guwahati, 781101 India; 3 Advanced Pediatrics Centre, PGIMER, Chandigarh, 160012 India; 4 Biology and Serology Division, Department of Home (Police), Govt. of MP, Regional Forensic Science Laboratory, Bhopal, 462003 India; 5 DNA Division, State Forensic Science Laboratory, Jaipur, Rajasthan 302016 India

**Keywords:** Biological techniques, Genetics

## Abstract

Kinship analysis in forensics is based on the calculation of the respective kinship indices. However, this calculation is only possible when the subject under identification has been associated with a particular population, whose allele frequency data is available for the particular set of STR markers used in the forensic practices. In the case of mass disasters, where a large number of individuals are to be identified, gathering the population frequency data and calculating the kinship indices can be an intricate process which requires a lot of time and huge resources. The new method of allele matching cut off score (AMCOS) developed in this study is based on the allele sharing approach. This approach simply refers to the number of shared alleles (1 or 2) between the two individuals; also known as identical by state (IBS) alleles which might have been inherited from a recent common ancestor in which the alleles are identical by descendent (IBD). In case of mass disasters, this method can be used to narrow down the number of pairs (dead and alive) to be matched for kinship without using the allele frequency data. The results obtained from this method could further be confirmed by LR based method, which uses the allele frequency data of the respective population of the pairs being tested for kinship. AMCOS method has been tested for its sensitivity, specificity and various other statistical parameters and has shown promising values for the same in various types of kinship analyses. This ascertains the authenticity and potential use of this method in forensic practice but only after its validation in a larger sample size. AMCOS method has been tested on siblings and grandparent-grandchildren by using autosomal and X-STR markers both, as the reference samples from the parents cannot always be available for the identification. The present study also compared the results shown by the autosomal and X-STR markers in siblings and grandparent-grandchildren identification, thereby suggesting the use of better set of markers on the basis of obtained values of various statistical parameters.

## Introduction

Sib ship analysis plays a vital role in identification of an individual for civil and criminal law cases and for searching a missing person when the parents are absent or dead^1^. In situations, where parentage (family trio) analysis is not feasible, DNA comparison with an alleged sibling could solve the purpose of identification. Since there are no obligatory alleles between the siblings which can help in excluding the case with absolute certainty, sibship analyses are more complicated^2^. It is not possible to eliminate sibship with confidence by using the genetic markers if only siblings are available for the study^3^.

According to Mendelian genetics law, full siblings acquire the alleles from their parents. Probabilities that a full sibling will share 0, 1, or 2 alleles IBD is ¼, ½, and ¼^4^. Various studies have been conducted to develop and access the validity of the sibling comparison test. Wenk et al.^3^ used three independent polymorphic VNTRs loci to establish a sibling comparison test^3^.

STR multiplex markers are the predominantly used for human identification^5^. Multiplex STRassays have been evaluated for their use in pairwise kinship analysis^2,6^. A study has been conducted on 9, 12 and 15-STR markers to develop a method for sibling identification^7^.

The study of kinship analysis requires the analysis of IBD alleles^8^, as the research related to genetic relatedness has always been linked to the root concept of IBD. Previously reported studies on sib ship analysis have also been based on the idea of IBD^9–12^. By combining the IBD method with the IBS information, the inference of genetic relatedness between the individuals (in pedigree and/or in large population-based studies) has been reported by Stevens et al.^13^. Term IBS is used to describe the two identical alleles at a locus between the two individuals who do not share a recent common ancestry. This method was proposed by Chakraborty and Jin (1983) for the inference of a pairwise relationship^14^. On the contrary, IBD describes two identical alleles that share a common ancestry. Two individuals who share 1 or 2 alleles IBS at a given locus may have inherited the alleles from a recent common ancestor in which the alleles were IBD^13^.

Yuan et al.^4^ reported the application of autosomal STR loci using the IBS method, and a discriminant function algorithm was also studied for their utility in Sibling identification. It was concluded from the study that STRs with higher discrimination power (PD) values should be selected when additional autosomal markers are required for full sibling identification. Moreover, discriminant analysis with IBS was reported to be highly useful for the full sibling test^4^.

Inferring a biological relationship from pairwise genetic data in loci is based on the allele frequencies of the observed alleles shared by the pairs of the individuals and on the probability equations for genotype combinations^3,15^. Likelihood ratio (LR) is calculated by using the frequency data to express the probability ratio of the relatives to the non-relatives. However, in some cases where the population frequencies of alleles may be unknown, or the ethnic origins may be unclear for foreign individuals, the LR based method fails to infer the relationship between the two individuals^15^.

In this study, we have used the allele sharing approach. This simply refers to the number of shared alleles (1 or 2) between the two individuals, also known as IBS alleles which might have been inherited from a recent common ancestor in which the alleles must have been IBD^13,16^ and developed a new method, named as AMCOS. The utility of this method was checked in the siblings and grandparent-grandchildren (GP-GC) identification cases. We applied AMCOS on the sibling and the grandparent-grandchildren data obtained from the most frequently used autosomal and relatively newer X-STR markers. The reason for choosing X-STR markers was their increasing popularity and their promising performance in kinship testing^17,18^. These X-STRs have also been recommended for use in certain pedigree analyses, which are reported to be indistinguishable by autosomal STRanalysis^8^. Also, X-chromosome marker typing has the ability to utilize short amplicons and ease of analysis over mtDNA, which is an intricate process^19,20^. These Characteristics makes X–STR markers suitable for the study of degraded samples from the mass disasters^21^. Autosomal STR analysis was chosen because the unlinked biallelic markers are being used worldwide as a standard practice in forensic laboratories for the last two decades^22,23^. The present study based on AMCOS values will give us a cut-off score/value based on IBS allele matches (which could be IBD also) between the siblings and grandparents-grand children. The cut-off score/value can be used to shortlist the number of pairs (deceased and their kin) to be matched and confirm the sibship and GP-GC identification in cases of any mass disaster or natural calamity. This AMCOS based method would help the analyst to save time and resources by short listing the number of individuals to be matched for kinship establishment in Disaster Victim Identification (DVI), which may further be validated by LR based approach. Once studied and validated in a larger sample size, this method could serve as a promising approach and can be used alone for the establishment of kinship.

## Results

### Brother-Sister (B-S)analysis

#### B-S analysis by autosomal STRs

Part 1: analysis of one allele matching (OAM) score.

Part 2: analysis of two allele matching (TAM) score.

##### Part 1: analysis of OAM score

**Figure 1:**
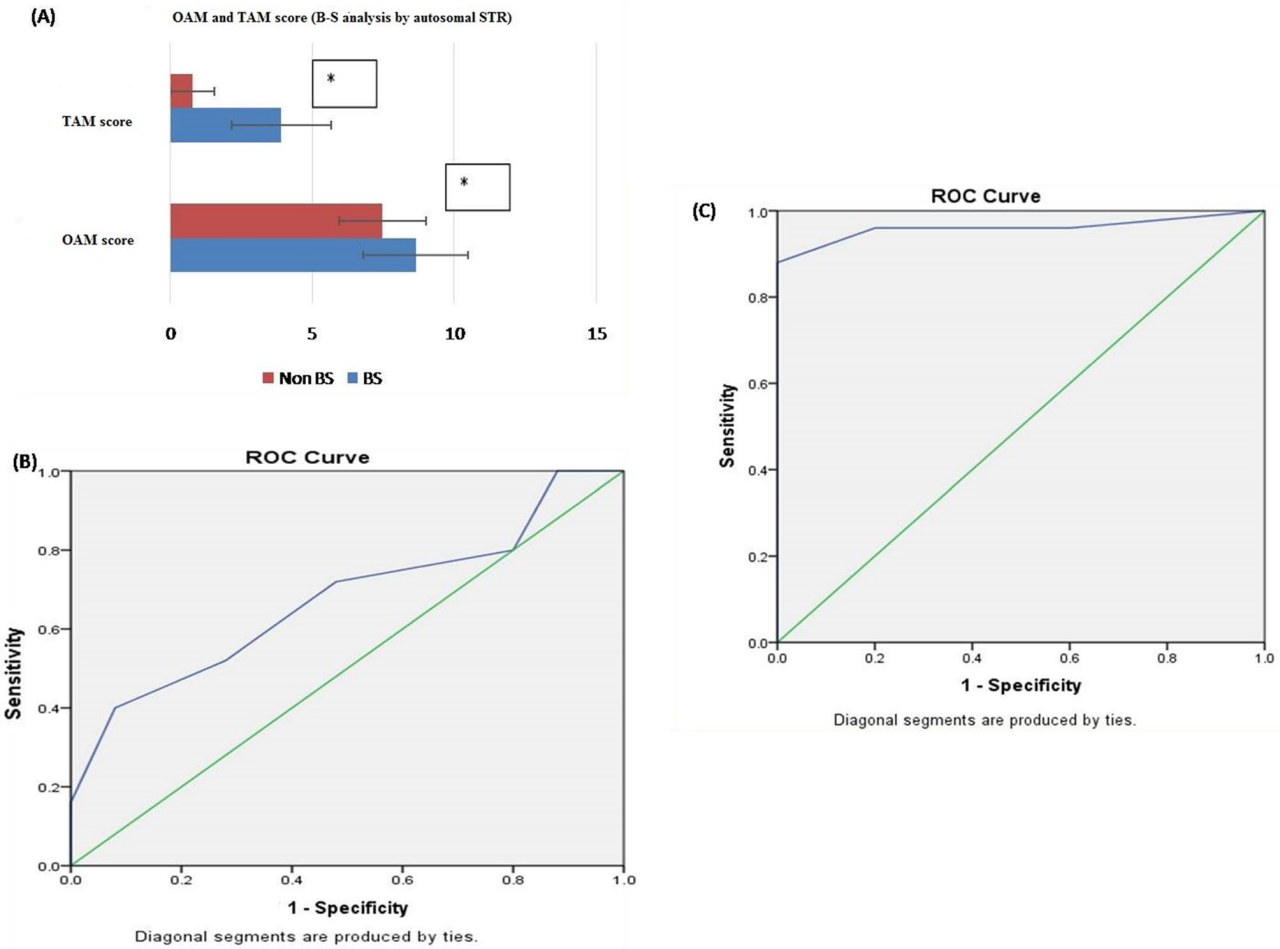
(**A**) OAM and TAM score for B-S analysis by autosomal STR. based on OAM and TAM score, statistically significant difference between B-S (related) and non-B-S (unrelated) groups was seen with Autosomal STR. The average TAM score for the B-S group is 3.92 ± 1.754 (SD), and for the non-B-S group average, TAM is 0.80 ± 0.764 (SD). Whereas the average OAM score for the B-S group is 8.64 ± 1.846 (SD) and for the non-B-S group is 7.48 ± 1.531 (SD). Figure 1 (**A–C**) obtained from SPSSsoftware version 22.0 (Available for download at: https://www.ibm.com/support/pages/downloading-ibm-spss-statistics-22). *Statistically significant difference ( p < 0.05). (**B**) ROC curve (AUC 67%), showing the performance of OAM score as a screening test for B-S pairs by autosomal STR. (**C**) ROC curve (AUC 96%), showing the performance of TAM score as a screening test for B-S pairs by autosomal STR.

- Based on OAM score, statistically significant difference between B-S (related) and non-B-S (unrelated) group was seen. The average OAM score for the B-S group is 8.64 ± 1.846 and for the non-B-S group is 7.48 ± 1.531 (Fig. 1A).
- Receiver operator curve (ROC) was plotted to evaluate the performance of OAM score as a screening test for B-S kinship. The area under the curve (AUC), which represents the accuracy of the test in discriminating the B-S cases from non B-S cases, was 67% (Fig. 1B).
- On the basis of calculated Yauden’s index, optimal AMCOS was chosen from the coordinates of the ROC curve (Table 1). Highest Yauden’s index was shown by the allele matching cut off score of 8.5. However the cut off score cannot be taken in decimals, so it was rounded off to 9. This AMCOS of 9 was further evaluated for sensitivity, Specificity, Positive likelihood ratio (LR +), negative likelihood ratio (LR −), Positive predictive value (PPV), negative predicative value (NPV) and accuracy of test.

**Table 1:** Sensitivity and 1-specificity values at different allele matching scores (AMS), calculated based on the ROC curve (Coordinates of the curve) in B-S kinship analysis by Autosomal STR.

Test result variable(s): OAM score		
OAM score^s^	Sensitivity (Y-axis coordinates)	1 – specificity (X-axis coordinates)
3.00	1.000	1.000
4.50	1.000	0.960
5.50	1.000	0.880
6.50	0.800	0.800
7.50	0.720	0.480
8.50	0.520	0.280
9.50	0.400	0.080
10.50	0.160	0.000
11.50	0.040	0.000
13.00	0.000	0.000

The smallest OAM cutoff score value is the minimum observed test value minus 1, and the most considerable cutoff value is the maximum perceived test value plus 1. All the other cutoff values are the averages of two consecutive ordered observed test values.

*Observation:* The sensitivity and specificity of the test with AMCOS of 9 were found to be 52 and 72%, respectively. The predictive values for positive and negative predictions were found to be 65 and 60%, respectively, and the overall accuracy of the test was found to be 62% (Table 11).

##### Part 2: analysis of TAM score

- Based on TAM score, statistically significant difference between B-S (related) and non-B-S (unrelated) group was seen. The average TAM score for the B-S group is 3.92 ± 1.754 (SD), and for the non-B-S it is 0.80 ± 0.764 (SD) (Fig. 1A).
- ROC curve was plotted to evaluate the performance of TAM score as a screening test for B-S kinship. The AUC, which represents the accuracy of the test in in discriminating the true cases (B-S cases) from non B-S, was 96% (Fig. 1C).
- On the basis of calculated Yauden’s index, optimal AMCOS was chosen from the coordinates of the ROC curve (Table 2). Highest Yauden’s index was shown by the allele matching cut off score of 2.5. However the cut off score cannot be taken in decimals, so it was rounded off to 3. This AMCOS of 3 was further evaluated for sensitivity, Specificity, Positive likelihood ratio (LR +), negative likelihood ratio (LR −), Positive predictive value (PPV), negative predicative value (NPV) and accuracy of test.

*Observation:* The sensitivity and specificity of the test with AMCOS of 3 were found to be 92 and 100 %, respectively. The predictive values for positive and negative predictions were found to be 100 and 92.59%, respectively, and the overall accuracy of the test was found to be 96% (Table 11).

**Table 2:** Sensitivity and 1-specificity values at different AMS, calculated based on the ROC curve (Coordinates of the curve) in B-S kinship analysis by autosomal STR.

Test result variable(s): TAM score		
TAM score^s^	Sensitivity (Y-axis coordinates)	1—Specificity (X-axis coordinates)
− 1.00	1.000	1.000
0.50	0.960	0.600
1.50	0.960	0.200
2.50	0.920	0.000
3.50	0.520	0.000
4.50	0.320	0.000
5.50	0.120	0.000
7.00	0.080	0.000
9.00	0.000	0.000

The smallest TAM cutoff score value is the minimum observed test value minus 1, and the most considerable cutoff value is the maximum perceived test value plus 1. All the other cutoff values are the averages of two consecutive ordered observed test values.

#### B-S analysis by X-STR

**Figure 2:**
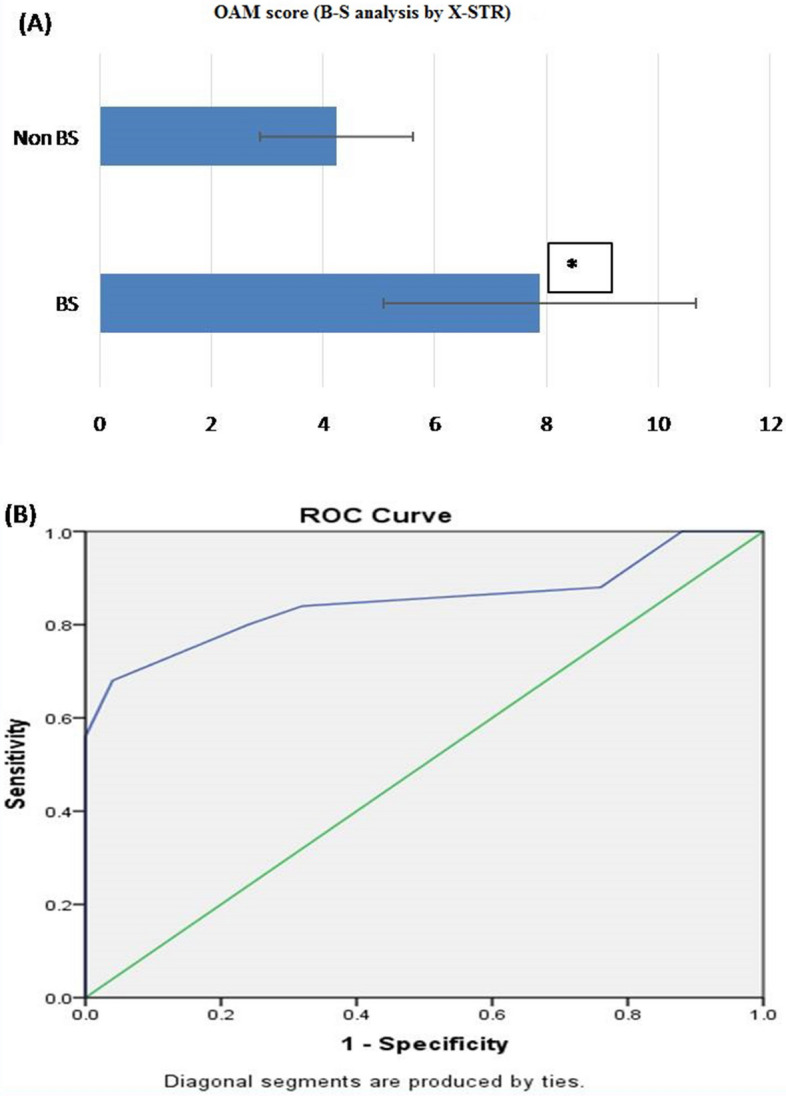
(**A**) OAM score for B-S analysis by X-STR. Based on the OAM score, statistically significant difference between B-S (related) and non-B-S (unrelated) groups was seen with X-STR. Average OAM score for B-S group is 7.88 ± 2.075 (SD), whereas for the non-B-S group is 4.24 ± 1.363 (SD). Figure 2 (A &B) obtained from SPSS software version 22.0 (Available for download at: https://www.ibm.com/support/pages/downloading-ibm-spss-statistics-22). *Statistically significant difference (p < 0.05). (**B**) ROC curve (AUC 85%), showing the performance of OAM score as a screening test for B-S pairs by X-STR.

- Based on OAM score, statistically significant difference between B-S (related) and non-B-S (unrelated) group was seen. Average OAM score for B-S group is 7.88 ± 2.075 (SD). Whereas, for the non-B-S group is 4.24 ± 1.363 (SD) (Fig. 2A).

**Table 3:** Sensitivity and 1-specificity values at different AMS, calculated based on the ROC curve (Coordinates of the curve) in B-S kinship analysis by X-STR.

Test result variable(s): maternal OAM score		
OAM score	Sensitivity (Y-axis coordinates)	1—Specificity (X-axis coordinates)
1.00	1.000	1.000
2.50	1.000	0.880
3.50	0.880	0.760
4.50	0.840	0.320
5.50	0.800	0.240
6.50	0.680	0.040
7.50	0.560	0.000
8.50	0.520	0.000
9.50	0.360	0.000
10.50	0.160	0.000
11.50	0.080	0.000
13.00	0.000	0.000

The smallest OAM cutoff value is the minimum observed test value minus 1, and the largest cutoff value is the maximum observed test value plus 1. All the other cutoff values are the averages of two consecutive ordered observed test values.
- ROC curve was plotted to evaluate the performance of OAM score as a screening test for B-S kinship. The AUC, which represents the accuracy of the test in discriminating the B-S cases from non B-S case, was 85%, (Fig. 2B).
- On the basis of calculated Yauden’s index, optimal AMCOS was chosen from the coordinates of the ROC curve (Table 3). Highest Yauden’s index was shown by the allele matching cut off score of 5.5. However the cut off score cannot be taken in decimals, so it was rounded off to 6. This AMCOS of 6 was further evaluated for sensitivity, Specificity, Positive likelihood ratio (LR +), negative likelihood ratio (LR −), positive predictive value (PPV), negative predicative value (NPV) and accuracy of test.

*Observation:* The sensitivity and specificity of the test with AMCOS of 6 were found to be 80 and 76%, respectively. The predictive values for positive and negative predictions were found to be 76.92 and 79.17%, respectively, and the overall accuracy of the test was found to be 78% (Table 11).

### Brother-Brother (B-B) analysis

#### B-B analysis by autosomal STRs

**Figure 3:**
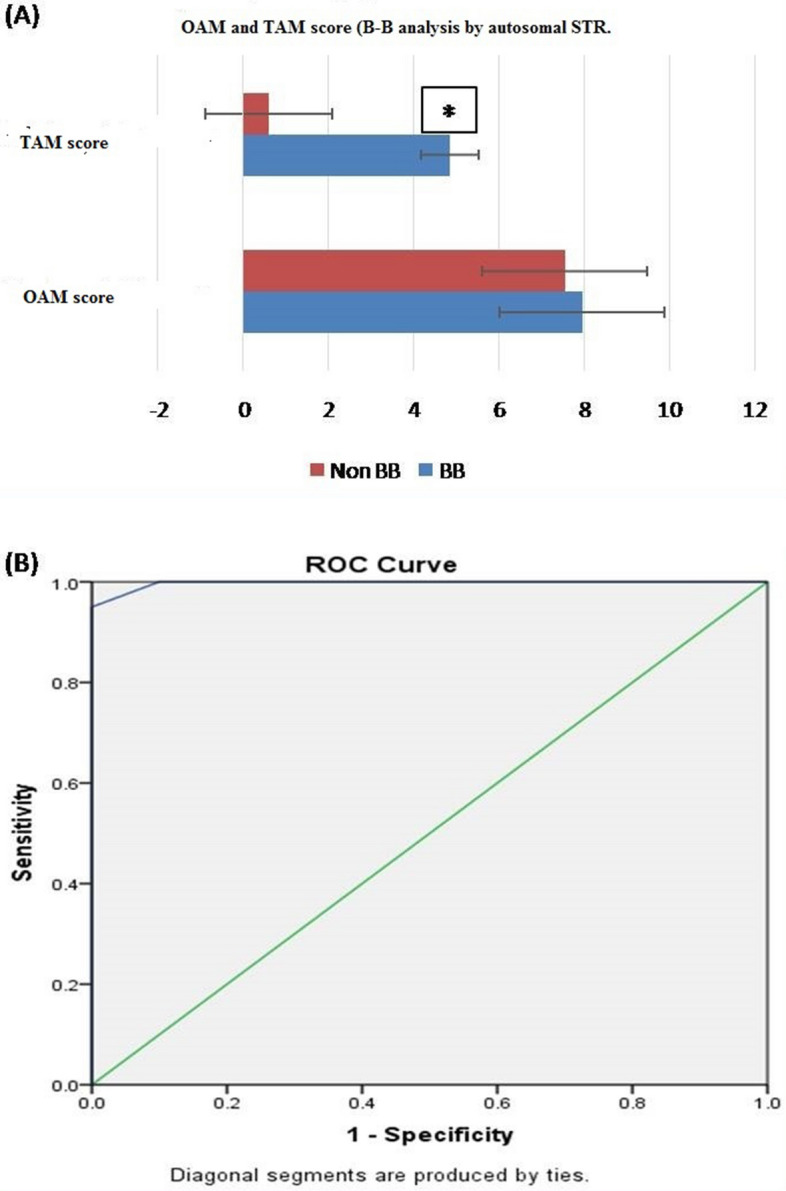
(**A**) TAM score for B-B analysis by autosomal STR. Based on the TAM score statistically significant difference between B-B (related) and non-B-B (unrelated) group was seen with autosomal STR. The average TAM score for B-B is 4.85 ± 1.496 (SD), whereas, for the non-B-B group, the average TAM is 0.60 ± 0.681 (SD). Figure 3 (A & B) obtained from SPSS software version 22.0 (Available for download at: https://www.ibm.com/support/pages/downloading-ibm-spss-statistics-22). *Statistically significant difference (p < 0.05). (**B**) ROC curve (AUC 99.7%), showing the performance of TAM score as a screening test for B-B pairs by autosomal STR.

- OAM and TAM scores, both were analyses but only TAM score showed statistically significant difference between B-B (related) and non-B-B (unrelated) group. The average TAM score for B-B is 4.85 ± 1.496 (SD). Whereas, for the non-B-B group, the average TAM is 0.60 ± 0.681 (SD) (Fig. 3A).
- ROC curve was plotted to evaluate the performance of TAM score as a screening test for B-B kinship, the area under the curve, which represents the accuracy of the test in discriminating the B-B cases) from non B-B cases, was 99.77% (Fig. 3B).

**Table 4:** Sensitivity and 1-specificity values at different AMS, calculated based on the ROC curve (Coordinates of the curve) in B-B kinship analysis by autosomal STR.

Test result variable(s): TAM score		
TAM score^a^	Sensitivity Y-axis coordinates	1—specificity X-axis coordinates
− 1.00	1.000	1.000
0.50	1.000	0.500
1.50	1.000	0.100
2.50	0.950	0.000
3.50	0.800	0.000
4.50	0.600	0.000
5.50	0.350	0.000
6.50	0.100	0.000
7.50	0.050	0.000
9.00	0.000	0.000

The smallest TAM score cutoff value is the minimum observed test value minus 1, and the most considerable cutoff value is the maximum perceived test value plus 1. All the other cutoff values are the averages of two consecutive ordered observed test values.
- On the basis of calculated Yauden’s index, optimal AMCOS was chosen from the coordinates of the ROC curve (Table 4). Highest Yauden’s index was shown by the allele matching cut off score of 2.5. However the cut off score cannot be taken in decimals, so it was rounded off to 3. This AMCOS of 3 was further evaluated for sensitivity, Specificity, Positive likelihood ratio (LR +), negative likelihood ratio (LR −), positive predictive value (PPV), negative predicative value (NPV) and accuracy of test.

*Observation:* The sensitivity and specificity of the test with AMCOS of 3 were found to be 95 and 100%, respectively. The predictive values for positive and negative predictions were found to be 100 and 95.24%, respectively, and the overall accuracy of the test was found to be 97.5% (Table 11).

#### B-B analysis by X STR

**Figure 4:**
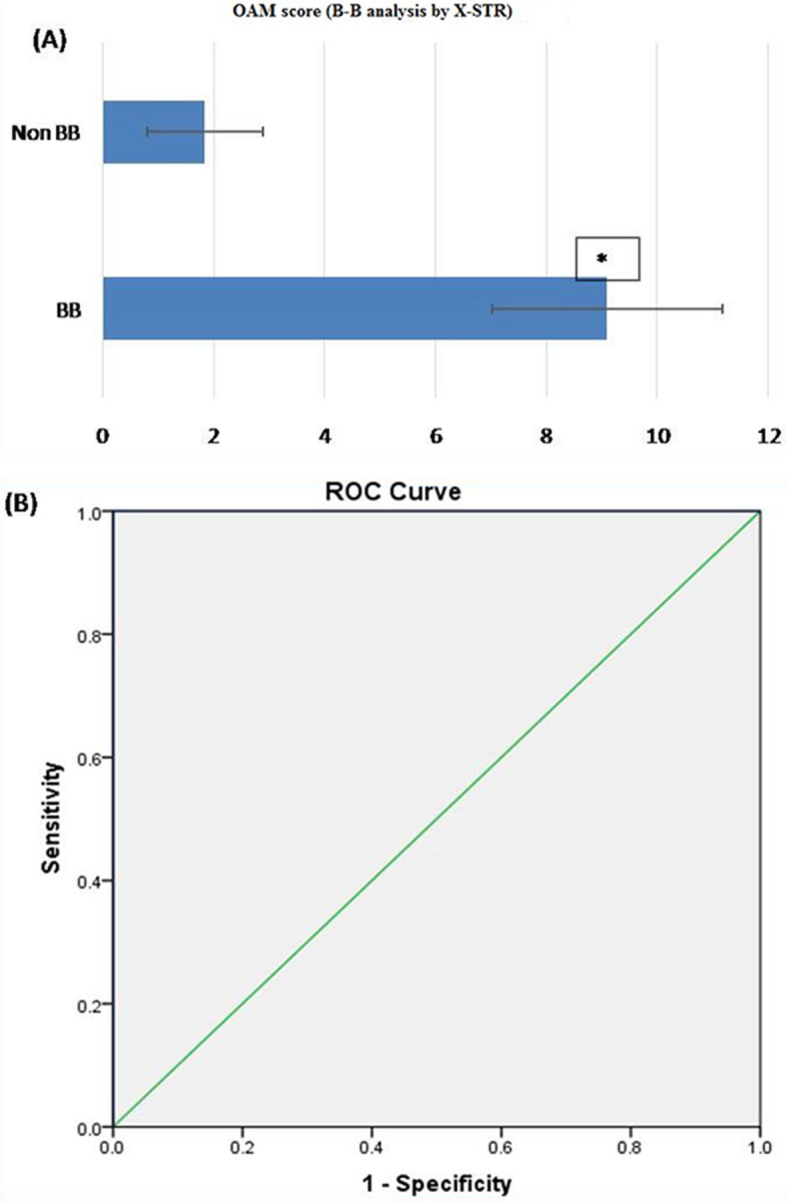
(**A**) OAM score for B-B analysis by X-STR. Based on OAM score, statistically significant difference between B-B and non-B-B group was seen with X-STR. The Average OAM score for the B-B group is 9.1 ± 2.075 (SD), whereas for the non-B-B group average OAM score is 1.85 ± 0.040 (SD). Figure 4 (A & B) obtained from SPSS software version 22.0 (Available for download at: https://www.ibm.com/support/pages/downloading-ibm-spss-statistics-22). *Statistically significant difference ( p < 0.05). (**B**) ROC curve (AUC 100%), showing the performance of OAM score as a screening test for B-B pairs by X-STR.

- Based on OAM score, statistically significant difference between B-B (related) and non-B-B (unrelated) group was seen. The Average OAM score for the B-B group is 9.1 ± 2.075 (SD). Whereas for the non-B-B group average OAM score is 1.85 ± 0.040 (SD). (Fig. 4A).
- ROC curve was plotted to evaluate the performance of OAM score as a screening test for B-B kinship. The AUC, which represents the accuracy of the test in discriminating the B-B cases from non B-B cases, was 100%, (Fig. 4B).
- On the basis of calculated Yauden’s index, optimal AMCOS was chosen from the coordinates of the ROC curve (Table 5). Highest Yauden’s index was shown by the allele matching cut off score of 4.5. However the cut off score cannot be taken in decimals, so it was rounded off to 5. This AMCOS of 5 was further evaluated for sensitivity, Specificity, Positive likelihood ratio (LR +), negative likelihood ratio (LR −), positive predictive value (PPV), negative predicative value (NPV) and accuracy of test.

**Table 5:** Sensitivity and 1-specificity values at different AMS, calculated on the basis of the ROC curve (Coordinates of the curve) in B-B kinship analysis by X-STR.

Test result variable(s): OAM score		
OAM score	Sensitivity (Y-axis coordinates)	1—specificity (X-axis coordinates)
− 1.00	1.000	1.000
0.50	1.000	0.950
1.50	1.000	0.600
2.50	1.000	0.200
3.50	1.000	0.100
4.50	1.000	0.000
5.50	0.950	0.000
6.50	0.900	0.000
7.50	0.800	0.000
8.50	0.550	0.000
9.50	0.450	0.000
10.50	0.250	0.000
11.50	0.200	0.000
13.00	0.000	0.000

The smallest OAM cutoff score value is the minimum observed test value minus 1, and the most considerable cutoff value is the maximum perceived test value plus 1. All the other cutoff values are the averages of two consecutive ordered observed test values.

*Observation:* The sensitivity and specificity of the test with AMCOS of 5 were found to be 100 and 100%, respectively. The predictive values for positive and negative predictions were found to be 100 and 100%, respectively, and the overall accuracy of the test was found to be 100% (Table 11).

### Sister-Sister (S-S) analysis

#### S-S analysis by autosomal STR

**Figure 5:**
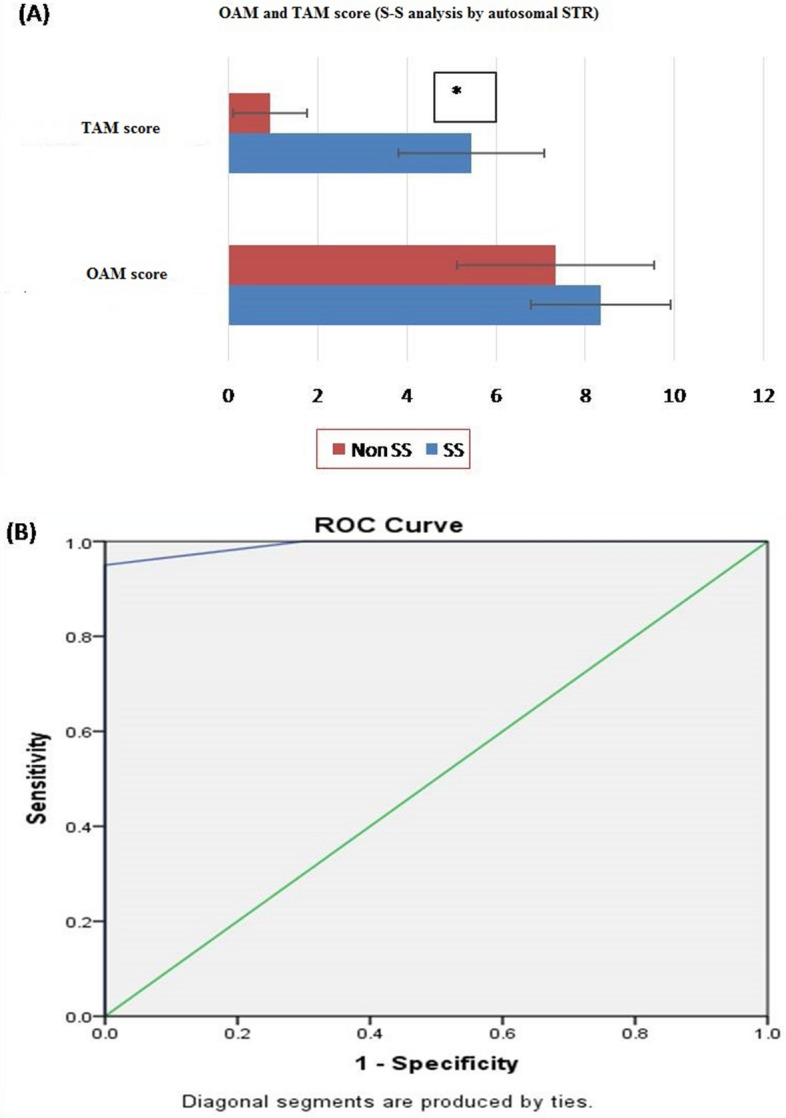
(**A**) TAM score for S–S analysis by autosomal STR. Based on the TAM score, statistically significant difference between S–S (related) and non-S–S (unrelated) groups was seen with autosomal STR. The Average TAM score for S–S is 5.45 ± 1.63 (SD), whereas, for the non-S–S group, the average TAM is 0.95 ± 0.326 (SD). Figure 5 (A & B) obtained from SPSSsoftware version 22.0 (Available for download at: https://www.ibm.com/support/pages/downloading-ibm-spss-statistics-22). *Statistically significant difference (p < 0.05). (**B**) ROC curve (AUC 99.2%), showing the performance of TAM score as screening test for S–S pairs by X-STR.

- OAM and TAM scores, both were analyzed but only TAM score showed statistically significant difference between S-S (related) and non-S-S (unrelated) group. The Average TAM score for S-S is 5.45 ± 1.63 (SD). Whereas, for the non-S-S group, the average TAM is 0.95 ± 0.326 (SD). (Fig. 5A).
- ROC curve was plotted to evaluate the performance of TAM score as a screening test for S-S kinship. The AUC, which represents the accuracy of the test in discriminating the true cases (S-S cases) from non S-S cases, was 99.2%, (Fig. 5B).
- On the basis of calculated Yauden’s index, optimal AMCOS was chosen from the coordinates of the ROC curve (Table 6). Highest Yauden’s index was shown by the allele matching cut off score of 2.5. However the cut off score cannot be taken in decimals, so it was rounded off to 3. This AMCOS of 3 was further evaluated for sensitivity, Specificity, Positive likelihood ratio (LR +), negative likelihood ratio (LR −), Positive predictive value (PPV), negative predicative value (NPV) and accuracy of test.

**Table 6:** Sensitivity and 1-specificity values at different AMS, calculated on the basis of the ROC curve (Coordinates of the curve) in S–S kinship analysis by autosomal STR.

Test result variable(s): TAM score		
TAM score^a^	Sensitivity (Y-axis coordinates)	1—Specificity (X-axis coordinates)
− 1.00	1.000	1.000
0.50	1.000	0.650
1.50	1.000	0.300
2.50	0.950	0.000
3.50	0.850	0.000
4.50	0.750	0.000
5.50	0.550	0.000
6.50	0.250	0.000
7.50	0.100	0.000
9.00	0.000	0.000

The smallest cutoff value is the minimum observed test value minus 1, and the most considerable cutoff value is the maximum observed test value plus 1. All the other cutoff values are the averages of two consecutive ordered observed test values.

*Observation:* The sensitivity and specificity of the test with AMCOS of 3 were found to be 95 and 100%, respectively. The predictive values for positive and negative predictions were found to be 100 and 95.2%, respectively, and the overall accuracy of the test was found to be 97.5% (Table 11).

#### S-S analysis by X-STR

**Figure 6:**
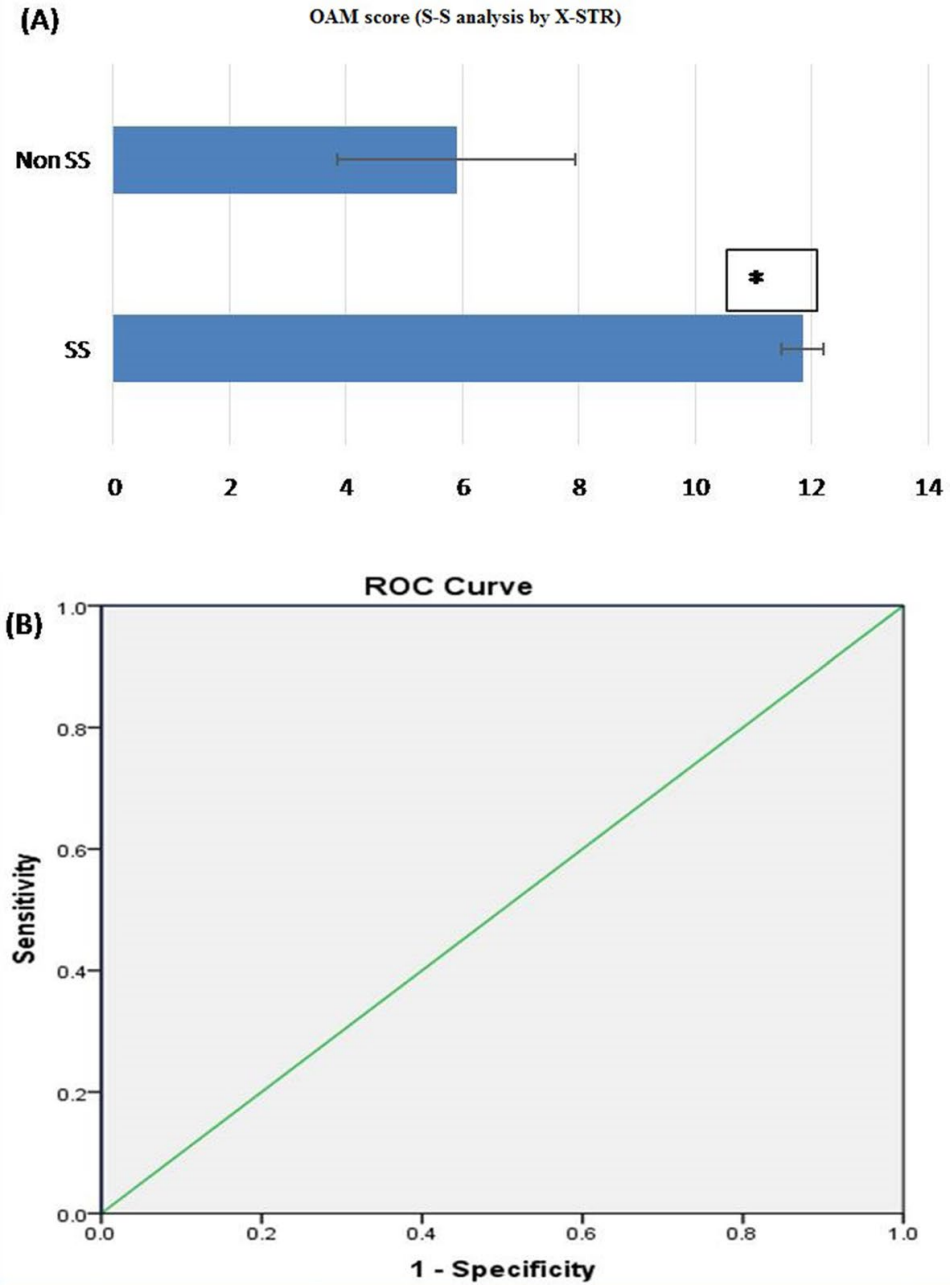
(**A**) OAM score for S–S analysis by X-STR: Based on OAM score, statistically significant difference between S–S (related) and non-S–S (unrelated) groups was seen with X-STR. The Average OAM score for S–S is 11.85 ± 0.366 (SD), whereas, for the non-S–S group, the average OAM is 5.90 ± 2.049 (SD). Figure 6 (A & B) obtained from SPSSsoftware version 22.0 (Available for download at: https://www.ibm.com/support/pages/downloading-ibm-spss-statistics-22). *Statistically significant difference (p < 0.05). (**B**) ROC curve (AUC 100%), showing the performance of OAM score as a screening test for S–S pairs by X-STR.

- Based on OAM score, statistically significant difference between S-S (related) and non-S-S (unrelated) group was seen. The Average OAM score for S-S is 11.85 ± 0.366 (SD). Whereas, for the non-S-S group, the average OAM is 5.90 ± 2.049 (SD) (Fig. 6A).
- ROC curve was plotted to evaluate the performance of OAM score as a screening test for S-S kinship. The AUC, which represents the accuracy of the test in discriminating the true cases (S-S cases) from non S-S cases, was 100%, (Fig. 6B).
- On the basis of calculated Yauden’s index, optimal AMCOS was chosen from the coordinates of the ROC curve (Table 7). Highest Yauden’s index was shown by the allele matching cut off score of 10.5. However the cut off score cannot be taken in decimals, so it was rounded off to 11. This AMCOS of 11 was further evaluated for sensitivity, Specificity, Positive likelihood ratio (LR +), negative likelihood ratio (LR −), Positive predictive value (PPV), negative predicative value (NPV) and accuracy of test.

**Table 7:** Sensitivity and 1-specificity values at different AMS, calculated on the basis of the ROC curve (Coordinates of the curve) in S–S kinship analysis by X-STR.

Test result variable(s): OAM score		
OAM score ^a^	Sensitivity (Y-axis coordinates)	1—specificity (X-axis coordinates)
1.00	1.000	1.000
3.00	1.000	0.900
4.50	1.000	0.800
5.50	1.000	0.550
6.50	1.000	0.450
7.50	1.000	0.150
8.50	1.000	0.100
9.50	1.000	0.050
10.50	1.000	0.000
11.50	0.850	0.000
13.00	0.000	0.000

The smallest OAM cutoff score value is the minimum observed test value minus 1, and the most considerable cutoff value is the maximum perceived test value plus 1. All the other cutoff values are the averages of two consecutive ordered observed test values.

*Observation:* The sensitivity and specificity of the test with AMCOS of 11 were found to be 100 and 100 %, respectively. The predictive values for positive and negative predictions were found to be 100 and 100%, respectively, and the overall accuracy of the test was found to be 100% (Table 11).

### Grandparent Grandchildren (GP-GC) analysis

#### GP-GC analysis by autosomal STR

**Figure 7:**
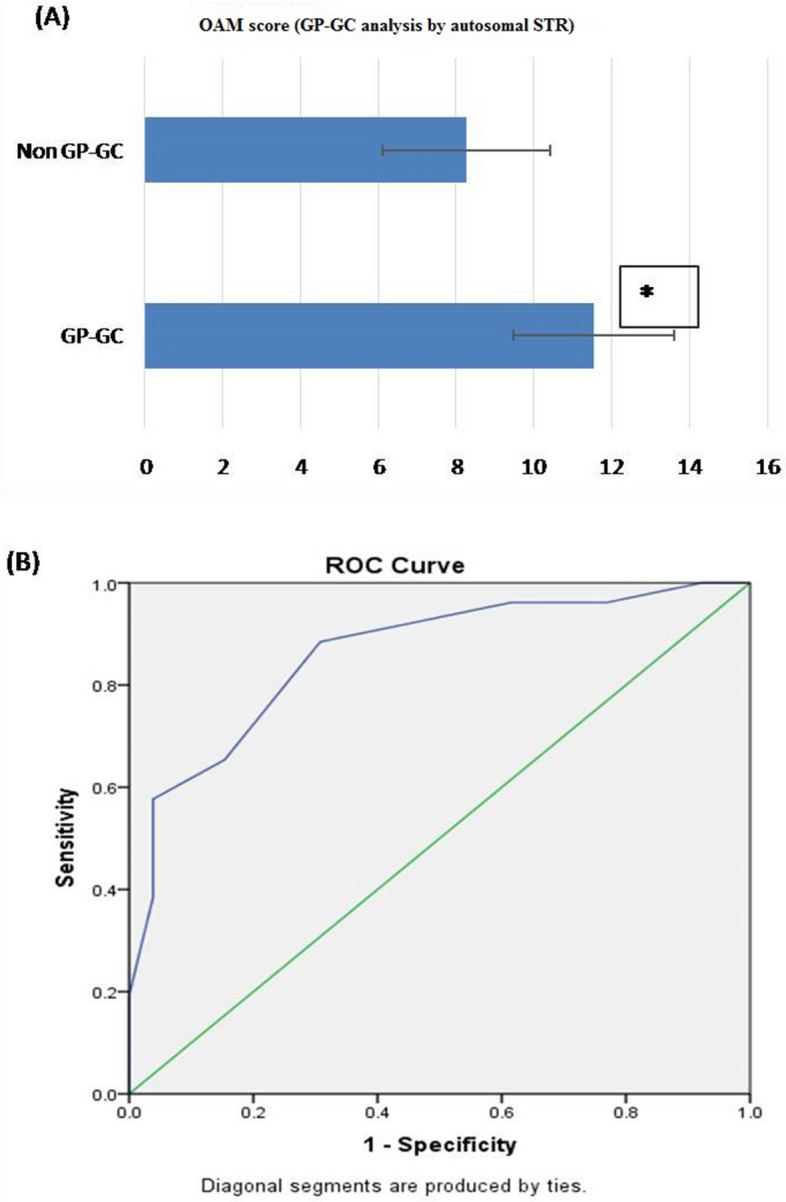
(**A**) OAM score for GP-GC analysis by autosomal STR: based on OAM score, statistically significant difference between GP-GC (related) and non-GP-GC (unrelated) groups was seen with autosomal STR. The Average OAM score for GP-GC is 11.54 ± 2.64 (SD), whereas, for the non-GP-GC group, the average OAM is 8.27 ± 2.146 (SD). Figure 7 (A & B) obtained from SPSS software version 22.0 (Available for download at: https://www.ibm.com/support/pages/downloading-ibm-spss-statistics-22). *Statistically significant difference (p < 0.05). (**B**) ROC curve (AUC 86%), showing the performance of OAM score as a screening test for GP-GC pairs by autosomal STR.

- Based on OAM score, statistically significant difference between GP-GC (related) and non-GP-GC (unrelated) group was seen. The Average OAM score for GP-GC is 11.54 ± 2.64 (SD). Whereas, for the non-GP-GC group, the average OAM is 8.27 ± 2.146 (SD) (Fig. 7A).
- ROC was plotted to evaluate the performance of OAM score as a screening test for GP-GC kinship, the AUC, which represents the accuracy of the test in discriminating the GP-GC cases from non GP-GC cases was 86%, (Fig. 7B).
- On the basis of calculated Yauden’s index, optimal AMCOS was chosen from the coordinates of the ROC curve (Table 8). Highest Yauden’s index was shown by the allele matching cut off score of 9.5. However the cut off score cannot be taken in decimals, so it was rounded off to 10. This AMCOS of 10 was further evaluated for sensitivity, Specificity, Positive likelihood ratio (LR +), negative likelihood ratio (LR −), Positive predictive value (PPV), negative predicative value (NPV) and accuracy of test.

**Table 8:** Sensitivity and 1-specificity values at different AMS, calculated based on the ROC curve (Coordinates of the curve) in GP-GC kinship analysis by autosomal STR.

Test result variable(s): OAM score		
OAM score^s^	Sensitivity (Y-axis coordinates)	1—specificity (X-axis coordinates)
3.00	1.000	1.000
4.50	1.000	0.962
5.50	1.000	0.923
6.50	0.962	0.769
7.50	0.962	0.615
8.50	0.923	0.462
9.50	0.885	0.350
10.50	0.654	0.154
11.50	0.577	0.038
12.50	0.385	0.038
13.50	0.192	0.000
15.00	0.000	0.000

^a^The smallest cutoff value is the minimum observed test value minus 1, and the largest cutoff value is the maximum observed test value plus 1. All the other cutoff values are the averages of two consecutive ordered observed test values.

*Observation:* The sensitivity and specificity of the test with AMCOS of 10 were found to be 85.2 and 65%, respectively. The predictive values for positive and negative predictions were found to be 74.19 and 85.71%, respectively, and the overall accuracy of the test was found to be 78.85% (Table 11).

#### GP-GC by X-STR analysis

Part 1: paternal grandparents.

Part 2: maternal Grandparents.

##### Part 1: paternal grandparents

**Figure 8:**
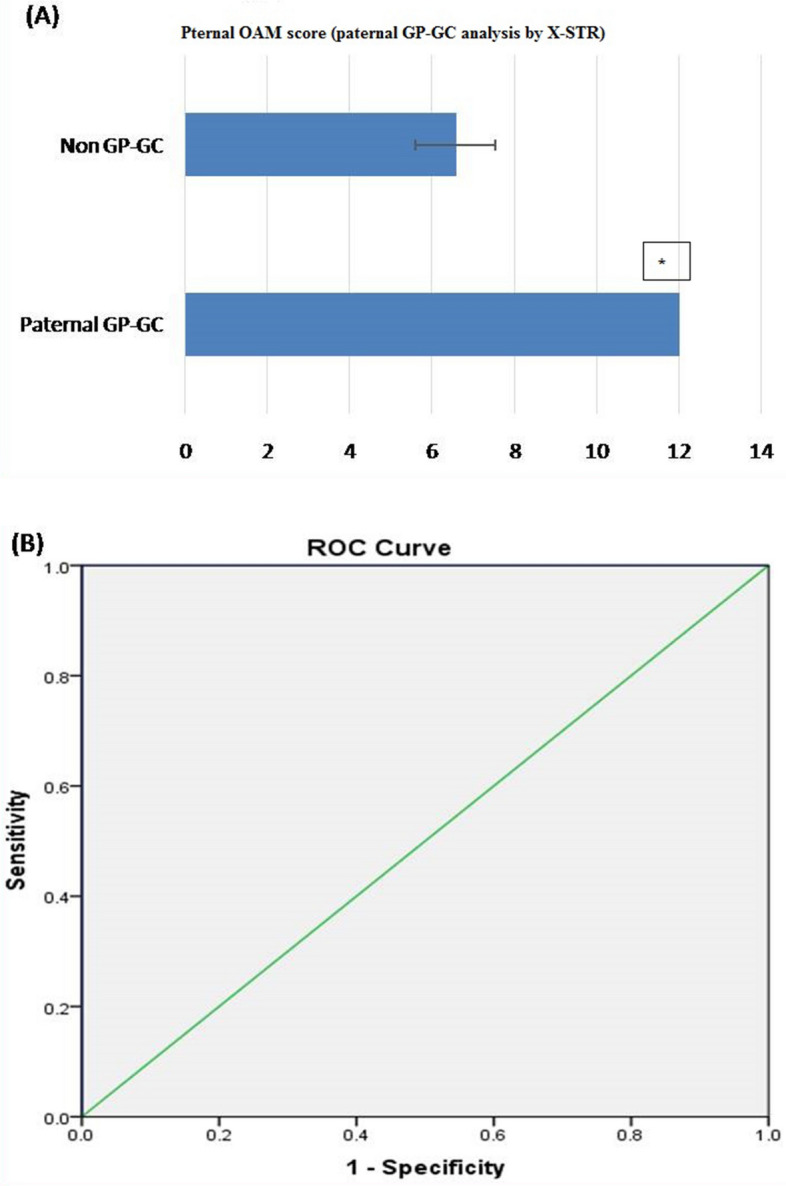
(**A**) OAM score for paternal GP-GC analysis by X-STR: Based on OAM score, statistically significant difference between paternal GP-GC (related) and non-GP-GC (unrelated) groups was seen with X-STR. The average OAM score for GP-GC is 12 ± 0.00 (SD), whereas, for the non-GP-GC group, the average OAM is 6.57 ± 0.976 (SD). Figure 8 (A & B) obtained from SPSSsoftware version 22.0 (Available for download at: https://www.ibm.com/support/pages/downloading-ibm-spss-statistics-22). *Statistically significant difference (p < 0.05). (**B**) ROC curve (AUC of 100%), showing the performance of OAM score as a screening test for Paternal GP-GC pairs by X-STR.

- Based on OAM score, statistically significant difference between Paternal GP-GC (related) and non-GP-GC (unrelated) group was seen. The average OAM score for GP-GC is 12 ± 0.00 (SD), whereas, for the non-GP-GC group, the average OAM is 6.57 ± 0.976 (SD) (Fig. 8A).
- ROC was plotted to evaluate the performance of OAM score as a screening test for paternal GP-GC kinship. The AUC, which represents the accuracy of the test in discriminating the GP-GC cases from non GP-GC cases, was 100%, (Fig. 8B).
- On the basis of calculated yauden’s index, optimal AMCOS was chosen from the coordinates of the ROC curve (Table 9). Highest Yauden’s index was shown by the allele matching cut off score of 10. This AMCOS of 10 was further evaluated for sensitivity, Specificity, Positive likelihood ratio (LR +), negative likelihood ratio (LR −), Positive predictive value (PPV), negative predicative value (NPV) and accuracy of test.

**Table 9:** Sensitivity and 1-specificity values at different AMS, calculated on the basis of the ROC curve (Coordinates of the curve) in paternal GP-GC kinship analysis by X-STR.

Test result variable(s): OAM score from paternal grandmother to granddaughter		
OAM score^a^	Sensitivity (Y-axis coordinates)	1—specificity (X-axis coordinates)
4.00	1.000	1.000
5.50	1.000	0.857
6.50	1.000	0.571
7.50	1.000	0.143
10.00	1.000	0.000
13.00	0.000	0.000

^a^The smallest OAM cutoff score value is the minimum observed test value minus 1, and the largest cutoff value is the maximum observed test value plus 1. All the other cutoff values are the averages of two consecutive ordered observed test values.

*Observation:* The sensitivity and specificity of the test with AMCOS of 10 were found to be 100 and 100 %, respectively. The predictive values for positive and negative predictions were found to be 100 and 100%, respectively, and the overall accuracy of the test was found to be 100% (Table 11).

##### Part 2: maternal grandparents

*Observation:* The sensitivity and specificity of the test with AMCOS of 6 were found to be 84.6 and 92.3%, respectively. The predictive values for positive and negative predictions were found to be 91.6 and 85.7%, respectively, and the overall accuracy of the test was found to be 88.46% (Table 11).

**Figure 9:**
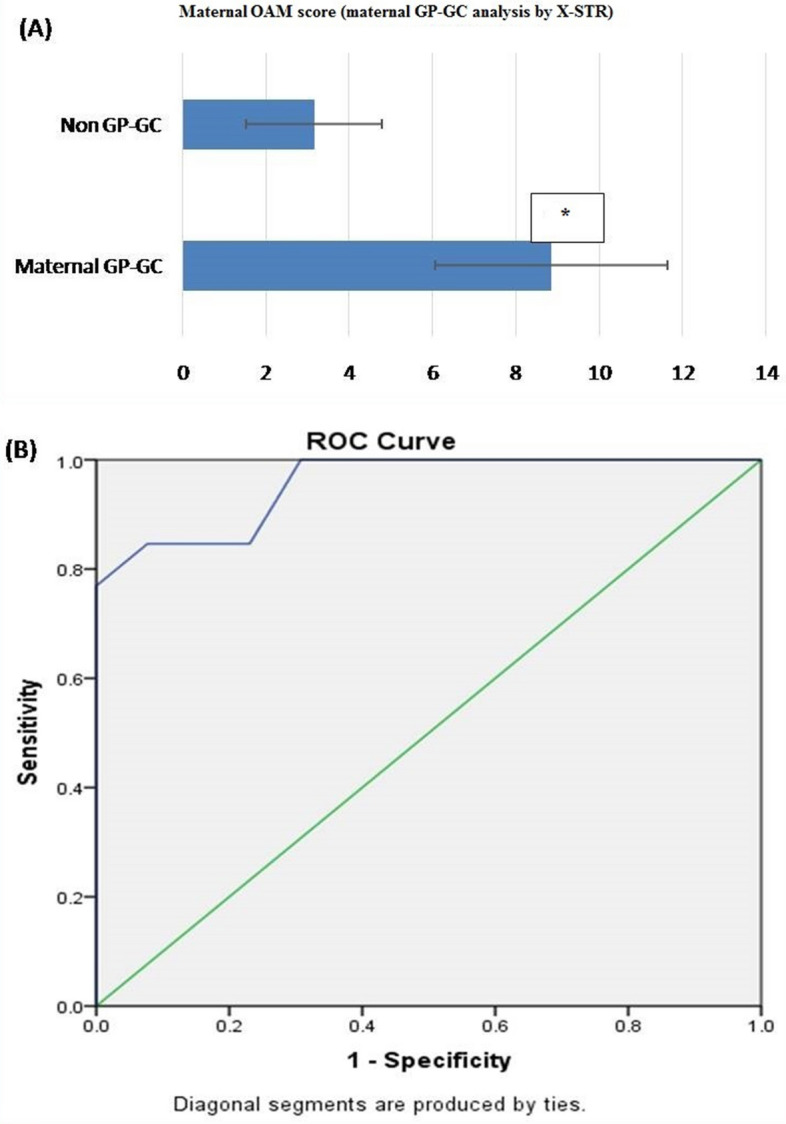
(**A**) OAM score for maternal GP-GC analysis by X-STR: Based on OAM score, statistically significant difference between maternal GP-GC (related) and non-GP-GC (unrelated) groups was seen with X-STR. The average OAM score for GP-GC is 8.85 ± 2.794 (SD), whereas, for the non-GP-GC group, the average OAM is 3.15 ± 1.625 (SD). Figure 9 (**A,B**) obtained from SPSS software version 22.0 (Available for download at: https://www.ibm.com/support/pages/downloading-ibm-spss-statistics-22). *Statistically significant difference (p < 0.05). (**B**) ROC curve (AUC 95.6%**)**, showing the performance of OAM score as a screening test for maternal GP-GC pairs by X-STR.

- Based on OAM score, statistically significant difference between maternal GP-GC (related) and non-GP-GC (unrelated) group was seen. The average OAM score for GP-GC is 8.85 ± 2.794 (SD). Whereas, for the non-GP-GC group, the average OAM is 3.15 ± 1.625 (SD) (Fig. 9A).
- ROC was plotted to evaluate the performance of OAM score as a screening test for maternal GP-GC kinship, the AUC, which represents the accuracy of the test in discriminating the GP-GC cases from non GP-GC cases, was 95.6%, (Fig. 9B).
- On the basis of calculated yauden’s index, optimal AMCOS was chosen from the coordinates of the ROC curve (Table 10). Highest Yauden’s index was shown by the allele matching cut off score of 5.5. Since the cut off score value can’t be taken in decimals, it was rounded off to 6. This AMCOS of 6 was further evaluated for sensitivity, Specificity, Positive likelihood ratio (LR +), negative likelihood ratio (LR −), Positive predictive value (PPV), negative predicative value (NPV) and accuracy of test.

**Table 10:** Sensitivity and 1-specificity values at different AMS, calculated on the basis of the ROC curve (Coordinates of the curve) in maternal GP-GC kinship analysis by X-STR.

Test result variable(s): maternal OAM score		
OAM score^a^	Sensitivity (Y-axis coordinates)	1 – Specificity (X-axis coordinates)
− 1.00	1.000	1.000
0.50	1.000	0.923
1.50	1.000	0.846
2.50	1.000	0.769
3.50	1.000	0.308
4.50	0.846	0.231
5.50	0.846	0.077
6.50	0.769	0.000
8.00	0.692	0.000
9.50	0.462	0.000
10.50	0.385	0.000
11.50	0.154	0.000
13.00	0.000	0.000

The smallest OAM cutoff score value is the minimum observed test value minus 1, and the largest cutoff value is the maximum observed test value plus 1. All the other cutoff values are the averages of two consecutive ordered observed test values.

**Table 11:** Sensitivity, specificity, positive predictive value (PPV), negative predictive value (NPV), positive likelihood ratio (LR +), negative likelihood ratio (LR-) and accuracy of AMCOS method in various types of kinship analyses.

Parameter	B-S			B-B		S-S		GP-GC		
	AUTO		X	AUTO	X	AUTO	X	AUTO	X	
	(OAM)	(TAM)	(OAM)	(OAM)	(OAM)	(TAM)	(OAM)	(OAM)	Paternal-GP (OAM)	Maternal- GP (OAM)
	AMCOS:9	AMCOS:3	AMCOS:6	AMCOS:3	AMCOS:5	AMCOS:3	AMCOS: 11	AMCOS:10	AMCOS:10	AMCOS:6
Sensitivity	52%	92%	80%	95%	100%	95%	100%	85.20%	100%	84.62%
	31.1–72.2%	73.97–99.02%	59.30–93.17%	75.13–99.87%	83.16–100%	75.13–99.87%	83.16–100%	69.85–97.55%	59.04–100%	54.55–98.08%
	(95% CI)	(95% CI)	(95% CI)	(95% CI)	(95% CI)	(95% CI)	(95% CI)	(95% CI)	(95% CI)	(95% CI)
Specificity	72%	100%	76%	100%	100%	100%	100%	65%	100%	92.31%
	50.61–87.93%	86.28–100%	54.87–90.64%	83.16–100%	83.16–100%	83.16–100%	83.16–100%	48.21–85.67%	59.04–100%	63.97–99.81%
	(95% CI)	(95% CI)	(95% CI)	(95% CI)	(95%CI)	(95%CI)	(95% CI)	(95%CI)	(95%CI)	(95%CI)
LR+	1.86	–	3.33	–	–	–	–	2.87	–	11
	0.89–3.86		1.62–6.88					1.59–5.20		1.65–73.3
	(95% CI)		(95% CI)					(95% CI)		(95% CI)
LR-	0.67	0.08	0.26	0.05	0	0.05	0	0.17	0	0.17
	0.41–1.07	0.02–0.30	0.12–0.59	0.01–0.34		0.01–0.3		0.06–0.50		0.05–0.6
	(95% CI)	(95% CI)	(95% CI)	(95% CI)		(95% CI)		(95% CI)		(95% CI)
PPV	65%	100%	76.92%	100%	100%	100%	100%	74.19%	100%	91.67%
	47.16–79.44%		61.76–87.81%					61.37–83.88%		62.26–98.65%
	(95% CI)		(95% CI)					(95% CI)		(95% CI)
NPV	60%	92.59%	79.17%	95.24%	100%	95.24%	100%	85.71%	100%	85.71%
	48.25–70.70%	76.79–97.93%	62.73–89.56%	74.75–99.27%		74.75–99.27%		66.75–94.72%		62.42–95.59%
	(95% CI)	(95% CI)	(95% CI)	(95% CI)		(95% CI)		(95% CI)		(95% CI)
Accuracy	62%	96%	78%	97.50%	100%	97.50%	100%	78.85%	100%	88.46%
	47.17–75.35%	86.29–99.51%	64.04–88.47%	86.84–99.94%	91.19–100%	86.84–99.94%	91.19–100%	65.30–88.94%	76.84–100%	69.55–97.55%
	(95% CI)	(95% CI)	(95% CI)	(95% CI)	(95% CI)	(95% CI)	(95% CI)	(95% CI)	(95% CI)	(95% CI)

Data showed as parameter value (95% confidence interval).

## Discussion

In case of mass disasters, the dead bodies or their mortal remains have to be identified and handed over to the concerned families to perform the last rights and for other civil matters like insurance, property and job claims. Enormous sample pairs have to be matched within a shorter time frame in such an emergency situation. There is a need for a rapid screening method which can screen out probable pairs, out of hundreds and thousands pairs of alive individuals (alleging to be kin of dead) and the dead bodies, and analyze relatedness for large number of sample pairs in such situations. The present study was designed with an aim to develop such a method. To avoid the wastage of time and resources, this study sets a standard allele match cut off score (AMCOS) as the minimum number of allele matches required considering the pairs for kinship (Table 11). AMCOS method is solely based on allele matches at different loci and does not require any allele frequency data. In this study, two set markers were used, autosomal and X-STRs, for the same set of kinship analyses (B-S, B-B, S-S, and GP-GC). Although, 12 X-STR markers used in this study exist in 4 linkage groups^24^, but each marker in the present study was matched individually for the screening of siblings and GP-GC. This was done to make the analysis unaffected by the population history with factors like population structure or small population size^25^. These factors affect the phenomenon of the linkage disequilibrium (LD) and thereby, the formation of linkage groups. Also the structure and patterns of LD are still unpredictable and poorly understood because of the interplay of regional recombination and demographic history, which is not well known^25,26^. As discussed above, the study remains unaffected by the population history; it can be used for any population and does not remain specific for a particular population. Theta (θ) correction, which is used as a measure for the effects of population subdivision (inbreeding), has not been employed in this study because of its very low value (much lower than 0.01) for most of the studied populations. Hence, the estimation of rarity of a DNA profile remains unaffected, whether substructure effects are considered or ignored^27,28^.

In B-S analysis by autosomal STR, a significant TAM score of 3 was found to be 92% sensitive with a specificity of 100% and accuracy of 96%. On the other hand, when B-S analysis was performed by X-STR, OAM of 6 was found to have sensitivity, specificity and accuracy of 80, 76 and 78% respectively. In B-B analysis by autosomal STR, the sensitivity, specificity and accuracy were 95, 100 and 97.5% respectively, while by X-STR the same set of B-B cases showed a sensitivity, specificity and accuracy of 100%. Similarly S-S analysis showed a sensitivity, specificity and accuracy of 100% with X-STRs and the same showed a sensitivity, specificity and accuracy of 95, 100 and 97.5% respectively, when autosomal STRs were used for the analysis. With a sensitivity, specificity and accuracy of 100% in paternal GP-GC cases and 84.6, 92.31 and 88.46% respectively in maternal GP-GC cases, X-STRs showed better values of statistical parameters in GP-GC cases as well.

Outcome of this study shows that the performance of X-STRs was found to be better in terms of statistical parameters like sensitivity, specificity in B-B, S-S, and GP-GC cases, while the NPV, and PPV did not show notable differences with both autosomal and X-STRs. Apart from B-S analysis by X-STR and GP-GC analysis by autosomal STR, where the PPV and NPV were found to be low. The autosomal STRoutperformed X-STRs in B-S identification cases and showed better values of all the statistical parameters. We tried to implicate the AMCOS method for GP-GC identification by autosomal STRanalysis, which otherwise is reported to be indistinguishable by the unlinked autosomal markers with LR based methods^8,29^. We observed significant values of statistical parameters using AMCOS method in GP-GC identification cases though comparatively lower values of PPV and NPV than X-STRs as mentioned above. The LR + and LR − values for all types of kinship analyses by autosomal and X-STRs were found to be in desired range (Table 11). To the best of the author's knowledge, AMCOS based method has never been used earlier to establish the kinship. The present study showed the successful implication of AMCOS method to screen out the probable siblings and GP-GC pairs. The results also support the potential use of this technique in forensic settings to identify the siblings and GP-GC, after its validation in a larger sample size. Based on the values of statistical parameters, the present study compares the results of X-STR and autosomal STRanalysis in the same samples (Table 11) and can be helpful in choosing the better set of markers for the above mentioned kinship analyses. It is emphasized again that the present study is a proof of concept study and needs to be conducted in a larger sample size of siblings and GP-GC both for further validation of its findings.

## Material and methodology

All methods were performed in accordance with the relevant guidelines and regulations of the institutional ethical committee of Post Graduate Institute of Medical Education, and Research. The study was commenced after taking ethical clearance from the internal ethical committee vide letter no: INT/IEC/2016/2409, Dated: Oct 4th 2016. 1 ml peripheral blood sample was withdrawn from the volunteer siblings and Grandparents-Grandchildren after taking the written informed consent. In case of children, written informed consent was taken either from parents or grandparents. The study was conducted at the Department of Forensic medicine, PGIMER, Chandigarh during 2014–2018. Total of 170 pairs, 50 B-S (25 test i.e. related pairs and 25 control i.e. unrelated pairs), 40 B-B (20 test and 20 control), 40 S-S (20 test and 20 control) and 40 GP-GC (20 test and 20 control), were studied. The kinship was confirmed verbally from the parents, siblings, and grandparents. Also, to ensure the sibship and grandparentage, we also followed the certainty threshold for likelihood ratios and selected the pairs with kinship indices between 100 and 1000(or > 1000)^30^. Since all the studied pairs hailed from the Sikh population of Punjab region of India, the population allele frequencies were calculated for the same population by using the GenAlEx software version 6.5^31^ and kinship (Sib ship and GP-GC) indices were calculated by using the X-STR data and FamLinkX software version 2.6^32^. X-STR data was used to confirm kinship because, as reported previously, unlinked autosomal STR markers are not efficient enough to distinguish the pedigrees like GP-GC^8,29^ with the likelihood ratio based method.

1. Extraction: DNA extraction was done by using the QIAamp DNA Blood Mini kit (Qiagen, Hilden, Germany) as per the manufacturer’s recommended protocol.

2. Quantification and amplification: The extracted DNAsamples were quantified using Quantifiler™ Human DNAquantification kit (ABI, ThermoFisher Scientific, US) as per the manufacturer’s recommended protocol. The samples were then amplified by using AmpFlSTR Identifiler Plus PCRamplification kit (Thermo Fisher Scientific, USA) for 15 autosomal markers (D8S1179, D21S11, D7S820, CSF1PO, D3S1358, TH01, D13S317, D16S539, D2S1338, D19S433, vWA, TPOX, D18S51, D5S818, and FGA) and Investigator Argus X-12 PCRAmplification Kit (Qiagen, Hilden, Germany) for 12 X STRmarkers (DXS10103, DXS8378, DXS7132, DXS10134, DXS10074, DXS10101, DXS10135, DXS7423, DXS10146, DXS10079, HPRTB, and DXS10148). Amplification of 500 pg (picogram) DNAwas performed according to the manufacturer’s recommended protocol of both the kits, except that the half of the reaction volume was used^33^.

3. Fragment analysis: Samples were run on genetic analyzer 3100 (Thermo Fisher Scientific, USA) using POP-4 with dye set G5. LIZ 500 (Thermo Fisher Scientific, USA) and BTO-550 (Qiagen, Hilden, Germany) were used as size standards for autosomal and X-STR analysis, respectively. Fragment analysis was performed as per the manufacturer’s recommended protocol. Obtained profiles were obtained and analyzed using profile quality parameters (data not shown).

4. Data analysis: Data was analyzed by using the Gene Mapper ID software version 3.2.1 (Available at http://tools.thermofisher.com/content/sfs/manuals/4352543.pdf). A peak detection threshold of 50 RFUs was used for allele designation. Alleles were designated on the basis of the number of allele’s repeats and in accordance with the guidelines of IFSG by the help of allelic ladders provided by the manufactures of both the kits, AmpFlSTR Identifiler Plus PCR Amplification Kit (Thermo Fisher Scientific, USA) and Investigator Argus X-12 PCR Amplification Kit (Qiagen, Hilden, Germany).

### Statistical analysis

1. Allele matching scores (AMS) of each related and unrelated pairs of B-S, B-B, S-S, and GP-GC were calculated. Methodology for the calculation of AMS, which was further used to calculate the AMCOS, in groups of siblings and GP-GC, is given in Supplementary Tables S1–S8.
2. Independent t-test or Mann–Whitney *U* test was applied on AMS of all the related and their respective unrelated pairs of all the groups (B-S, B-B, S-S, or GP-GC).
3. If the difference between the related and the unrelated groups (B-S, B-B, S-S, or GP-GC) was found to be significant (p < 0.05), that particular group was considered for further analysis.
4. All the statistical analysis was done by using SPSS software version 22.0 (Available for download at: https://www.ibm.com/support/pages/downloading-ibm-spss-statistics-22). Using the same software, receiver-operator curve (ROC) was drawn; area under this curve (AUC) depicted the efficiency of the test (in percentage) in discriminating the related cases from unrelated cases amongst all the groups (B-S, B-B, S-S, and GP-GC).
5. From the coordinates of the curve (ROC), the allele matching cut off score (AMCOS) was chosen by using the Youden’s index^34^. The AMCOS was selected for all the groups (B-S, B-B, S-S, and GP-GC) in a similar fashion (Tables 1, 2, 3, 4, 5, 6, 7, 8, 9, 10). NOTE: The AMCOS values with decimals were rounded off.
6. The allele matching score (AMS) of each studied pair of B-S, B-B, S-S and GP-GC groups (both related and unrelated) were compared against AMCOS (≥ or ≤ AMCOS) of the respective kinship groups.
7. Based on the comparison mentioned in point 6, a two by two (2 × 2) table was drawn for every individual pair (related and unrelated) of each group (B-S, B-B, S-S, and GP-GC) which showed the entire true positive (allele matching score ≥ AMCOS), true negative (allele matching score < AMCOS), false negative (related but allele matching score < AMCOS) and false-positive cases (unrelated but allele matching score ≥ AMCOS). The method was followed in all the pairs of both related and unrelated groups of (B-S, B-B, S-S, and GP-GC). Note: 2x2 tables not shown in results.
8. The obtained values from 2 × 2 tables in all the groups (B-S, B-B, S-S, and GP-GC) were further subjected to statistical analysis to evaluate the performance of AMCOS for parameters like sensitivity, specificity, positive likelihood ratio (LR +), negative likelihood ratio (LR −), negative predictive value (NPV), positive predictive value (PPV) and its accuracy (Table 11). The above-mentioned parameters were calculated by using the software MedCalc for windows version 15.0 (Available for download at https://www.medcalc.org/).
9. The sensitivity of the test discussed in the study refers to its ability to identify true positive cases of kinship, i.e., related individuals. In contrast, specificity defines the strength of the AMCOS method to detect the true negative cases of kinship, i.e., unrelated individuals alleged to be kins.
10. Positive likelihood ratio (LR +) is the probability that a positive test i.e. AMS ≥ AMCOS, would be expected in a related pair (True positive) divided by the probability that a positive test would be expected in unrelated pair (false positive), i.e. sensitivity/1-specificity^35^. Whereas negative likelihood ratio (LR-) is the probability of the related pair testing negative i.e. AMS ≤ AMCOS (false negative) divided by probability of unrelated pair testing negative (true negative) i.e. 1- sensitivity/specificity^35^. Note: The value of LR+ may vary from 1 to ∞. Larger the LR+, more informative would be the test. LR+ of 1 indicates that the test is useless and cannot discriminate related pairs from unrelated ones. Similarly the values of LR − may vary from 1 to 0. The smaller the LR-, more informative would be the test. LR- of 1 indicates that the test is useless and cannot discriminate related pairs from unrelated ones.
11. Positive predictive value (PPV) is the likelihood that the individuals who have been identified as related are actual kins (B-S, B-B, S-S or GP-GC). Similarly, negative predictive value (NPV) is the likelihood that the individuals who have been identified as unrelated are not related in reality.

### Ethical approval

All methods were performed in accordance with the relevant guidelines and regulations of the institutional ethical committee of Post Graduate Institute of Medical Education, and Research. The study was commenced after taking ethical clearance from the internal ethical committee vide letter no: INT/IEC/2016/2409, Dated: Oct 4th 2016. Peripheral blood sample was withdrawn from the volunteer siblings and Grandparents-Grandchildren after taking the written informed consent. In case of children, written informed consent was taken either from parents or grandparents.

## Supplementary Information

Supplementary Tables.
