# Supplementary material for: Development of a new screening method for faster kinship analyses in mass disasters: a proof of concept study
Source: Sci Rep. 2022 Nov 27;12:20372. doi: 10.1038/s41598-022-22805-w (PMC9701697; doi:10.1038/s41598-022-22805-w)
Supplement: Supplementary file 1 — Supplementary Tables. [file 41598_2022_22805_MOESM1_ESM.docx]

**Development of a new screening method for faster kinship analyses in mass disasters: A proof of concept study.**

Sonia Kakkar^1^, Phulen Sarma^2^, Inusha Panigrahi^3^, R.K. Kumawat^4^and Pankaj Shrivastava^5*^

^1^Department of Forensic Medicine, PGIMER, Chandigarh-160012, India. ^2^Department of Pharmacology, PGIMER, Chandigarh-160011, India. ^3^Advanced Pediatrics Centre, PGIMER, Chandigarh-462003, India. ^4^DNA Division, State Forensic Science Laboratory, Rajasthan, Jaipur-302016, India.

^5^Biology and Serology Division, Regional Forensic Science Laboratory, Department of Home (Police), Govt. of MP, Bhopal-462003 MP India

*Corresponding author: [pankaj.shrivastava@rediffmail.com](mailto:pankaj.shrivastava@rediffmail.com) Contact details: Phone No: +91-9424371946

**Note:** The methodology to calculate allele matching score (AMS) has been shown in only single pair of Brother- Sister (B-S), Brother-Brother (B-B), Sister-Sister(S-S) and Grandparent-Grandchildren (GP-GC). Using the same method, AMS was calculated in all the 50 pairs of B-S, and 40 pairs of B-B, S-S and GP-GCeach; and was used as mentioned in statistical analysis. Same set of related and unrelated pairs of all the groups were used in autosomal and X-STR analysis.

**Table S1: AMS calculation for B-S cases analyzed by autosomal STRs:** Both brother and sister receive 50% of their autosomal DNA from mother and 50% from father. After DNA profiles were generated; two allele matching (TAM) score and one allele matching (OAM) score both were seen for each test (related) and control (unrelated) pair of B-S.

**OAM score was seen.**

**Total OAM =6**

to

**TAM score was seen.**

**Total TAM =8**

to

| Locus | B1 | S1 |
| --- | --- | --- |
| D8S1179 | 13,14 | 13,14 |
| D21S11 | 28,31 | 28,31 |
| D7S820 | 10,11 | 9,11 |
| CSF1PO | 10,12 | 10,11 |
| D3S1358 | 16,16 | 15,16 |
| THO1 | 6,9 | 7,7 |
| D13S317 | 11,11 | 11,11 |
| D16S539 | 8,9 | 8,12 |
| D2S1338 | 22,23 | 17,23 |
| D19S433 | 13,14 | 13,14 |
| vWA | 14,17 | 14,17 |
| TPOX | 11,11 | 11,11 |
| D18S51 | 14,15 | 14,15 |
| D5S818 | 12,13 | 12,13 |
| FGA | 21,24 | 19,21 |
| AMEL | XY | XX |

**Table S2: AMS calculation for B-S cases analyzed by X- STRs:** Brother receives, its only, X chromosome from the mother and sister receives one of her X chromosomes from father and one from mother. After DNA profiles were generated, one (maternal) allele OAM score was seen for eachrelated and unrelated pairof B-S.

| LOCUS | B1 | S1 |
| --- | --- | --- |
| AMEL | XY | XX |
| DXS10101 | 19 | 18,19 |
| DXS8378 | 11 | 11,11 |
| DXS7132 | 16 | 13,15 |
| DXS10134 | 34 | 34,36 |
| DXS10074 | 17 | 17,19 |
| DXS10101 | 28.2 | 28.2,32 |
| DXS10135 | 22 | 19,22 |
| DXS7423 | 14 | 14,17 |
| DXS10146 | 28 | 28,30 |
| DXS10079 | 21 | 20,21 |
| HPRTB | 13 | 11,13 |
| DXS10148 | 18 | 18,24.1 |

**OAM score was seen.**

**Total OAM =11**

to

**Table S3: AMS Calculation for B-B cases analyzed by autosomal STRs:** Both the brothers receive 50% of their autosomal DNA from father and 50% from mother. After DNA profiles were generated, TAM score and OAM score both were seen for eachrelated and unrelated pair of B-B.

**TAM score was seen.**

**Total TAM =5**

to

| Locus | CP1B1 | CP1B2 |
| --- | --- | --- |
| D8S1179 | 10,12 | 14,16 |
| D21S11 | 29,32.2 | 29,30 |
| D7S820 | 8,12 | 8,12 |
| CSF1PO | 10,11 | 11,12 |
| D3S1358 | 16,17 | 16,17 |
| THO1 | 9,9.3 | 6,9 |
| D13S317 | 8,11 | 8,12 |
| D16S539 | 12,13 | 12,12 |
| D2S1338 | 19,24 | 18,23 |
| D19S433 | 14,14 | 14,14 |
| Vwa | 16,16 | 16,17 |
| TPOX | 11,11 | 8,11 |
| D18S51 | 11,14 | 10,13 |
| D5S818 | 12,14 | 12,14 |
| FGA | 21.2,23.2 | 21.2,23.2 |
| AMEL | XY | XY |

**OAM score was seen.**

**Total OAM =7**

to

**Table S4: AMS Calculation for the same set of B-B cases analyzed by X STRs:** Both the brothers receive their respective X chromosomes from their mother. So After the generation of DNA profiles, OAM score was seen for each related and unrelated pair of B-B.

| LOCUS | CP1B1 | CP1B2 |
| --- | --- | --- |
| AMEL | XY | XY |
| DXS10101 | 16 | 18 |
| DXS8378 | 11 | 11 |
| DXS7132 | 13 | 14 |
| DXS10134 | 32 | 32 |
| DXS10074 | 16 | 17 |
| DXS10101 | 28 | 33 |
| DXS10135 | 23 | 23 |
| DXS7423 | 15 | 15 |
| DXS10146 | 29 | 27 |
| DXS10079 | 19 | 20 |
| HPRTB | 13 | 14 |
| DXS10148 | 26.1 | 26.1 |

**OAMscore was seen.**

**Total OAM =5**

**Table S5**: **AMS Calculation for S-S cases analyzed by autosomal STRs:** similar to B-S and B-B inheritance, both the sisters will receive 50% of their autosomal DNA from mother and 50% from the father. After DNA profiles were generated, TAM score and OAM score both were seen for each related and unrelated pair of S-S.

| Locus | CP1S1 | CP1S2 |
| --- | --- | --- |
| D8S1179 | 10,10 | 10,16 |
| D21S11 | 29,30.2 | 28,29 |
| D7S820 | 11,14 | 10,11 |
| CSF1PO | 11,12 | 11,12 |
| D3S1358 | 15,16 | 15,16 |
| THO1 | 9,10 | 6,9 |
| D13S317 | 8,12 | 11,12 |
| D16S539 | 11,12 | 10,13 |
| D2S1338 | 18,19 | 18,19 |
| D19S433 | 14.2,15.2 | 14,14.2 |
| Vwa | 16,17 | 17,17 |
| TPOX | 8,9 | 8,9 |
| D18S51 | 12,16 | 12,16 |
| D5S818 | 10,12 | 10,13 |
| FGA | 19,20 | 20,25 |
| AMEL | XX | XY |

**OAM score was seen.**

**Total OAM =9**

to

**TAM score was seen.**

**Total TAM =5**

to

**Table S6:** AMS Calculation for the same set of S-S cases analyzed by X STRs: After DNA profiles were generated;OAM score was seen each related and unrelated S-S pair. Since it is known that the father transmits its X chromosome as a haplotype to all the daughters, we only considered OAM score for our analysis in order to cross-check the efficacy of our method.

| LOCUS | CP1B1 | CP1B2 |
| --- | --- | --- |
| AMEL | XX | XX |
| DXS10101 | 16,18 | 18,19 |
| DXS8378 | 10,13 | 10,12 |
| DXS7132 | 12,13 | 13,14 |
| DXS10134 | 35,37 | 35,36 |
| DXS10074 | 16,18 | 8,16 |
| DXS10101 | 30.2,32 | 29.2,30.2 |
| DXS10135 | 21,23 | 18,23 |
| DXS7423 | 15,15 | 14,15 |
| DXS10146 | 29,29 | 27,29 |
| DXS10079 | 15,20 | 20,20 |
| HPRTB | 12,13 | 12,13 |
| DXS10148 | 24.1,27.1 | 23.1,27.1 |

**OAM (paternal) score was seen.**

**Total OAM =12**

to

**Table S7: AMS calculation for GP-GC cases analyzed by autosomal STRs:**

In the case of GP-GC transmission, GC inherits 25% of their autosomal DNA from paternal and maternal GPs through their parents. After DNA profiles were generated, one OAM score was seen for each related and unrelated pair of GP-GC.

| Locus | CP1G1 | CP1C1 |
| --- | --- | --- |
| D8S1179 | 10,15 | 13,15 |
| D21S11 | 30,31.2 | 30,33.2 |
| D7S820 | 8,10 | 10,11 |
| CSF1PO | 9,12 | 11,12 |
| D3S1358 | 15,16 | 15,16 |
| THO1 | 8,9 | 6,9 |
| D13S317 | 11,12 | 9,11 |
| D16S539 | 9,11 | 11,12 |
| D2S1338 | 22,25 | 22,25 |
| D19S433 | 13,14 | 12,13 |
| VWa | 15,19 | 18,19 |
| TPOX | 9,11 | 8,11 |
| D18S51 | 11,19 | 17,19 |
| D5S818 | 8,11 | 8,11 |
| FGA | 20,20 | 22,26 |
| AMEL | XX | XX |

**OAM score was seen.**

**Total OAM =14**

to

**Table S8:AMS calculation for the same set of GP-GC (paternal and maternal) cases analyzed by X-STRs:**

In the case of X chromosomal DNA transmission, GC (boy and girl) receive 25% of their X chromosomal DNA from a maternal grandmother or maternal grandfather. And in the case of X chromosomal DNA inheritance from paternal grandparents, boy GC receives none from paternal grandparents, and girl GC receives 50% from paternal grandmother and none from paternal grandfather. After DNA profiles were generated, OAM score was seen for each related and unrelated pair of GP-GC..

| LOCUS | CP1G1 | CP1C1 |
| --- | --- | --- |
| AMEL | XX | XX |
| DXS10101 | 19,19 | 19,19 |
| DXS8378 | 11,12 | 11,11 |
| DXS7132 | 14,16 | 14,15 |
| DXS10134 | 34,35 | 34,39.3 |
| DXS10074 | 14,16 | 15,17 |
| DXS10101 | 28,31.2 | 29.2,30.2 |
| DXS10135 | 19,26 | 23,26 |
| DXS7423 | 15,17 | 15,15 |
| DXS10146 | 27,28 | 24,41.2 |
| DXS10079 | 16,21 | 18,19 |
| HPRTB | 14,14 | 12,15 |
| DXS10148 | 17,25.1 | 19,25.1 |

**OAM score was seen.**

**Total OAM =7**
